# Long distance biotic dispersal of tropical seagrass seeds by marine mega-herbivores

**DOI:** 10.1038/s41598-017-04421-1

**Published:** 2017-06-30

**Authors:** Samantha J. Tol, Jessie C. Jarvis, Paul H. York, Alana Grech, Bradley C. Congdon, Robert G. Coles

**Affiliations:** 1 Centre for Tropical Water and Aquatic Ecosystem Research (TropWATER), James Cook University, Cairns, Australia; 2 College of Science and Engineering, James Cook University, Cairns, Australia; 3 University of North Carolina Wilmington, Wilmington, United States of America; 4 ARC Centre of Excellence for Coral Reef Studies, James Cook University, Townsville, Australia

## Abstract

Terrestrial plants use an array of animals as vectors for dispersal, however little is known of biotic dispersal of marine angiosperms such as seagrasses. Our study in the Great Barrier Reef confirms for the first time that dugongs (*Dugong dugon*) and green sea turtles (*Chelonia mydas*) assist seagrass dispersal. We demonstrate that these marine mega-herbivores consume and pass in faecal matter viable seeds for at least three seagrass species (*Zostera muelleri*, *Halodule uninervis* and *Halophila decipiens*). One to two seagrass seeds per g DW of faecal matter were found during the peak of the seagrass reproductive season (September to December), with viability on excretion of 9.13% ± 4.61% (SE). Using population estimates for these mega-herbivores, and data on digestion time (hrs), average daily movement (km h) and numbers of viable seagrass seeds excreted (per g DW), we calculated potential seagrass seed dispersal distances. Dugongs and green sea turtle populations within this region can disperse >500,000 viable seagrass seeds daily, with a maximum dispersal distance of approximately 650 km. Biotic dispersal of tropical seagrass seeds by dugongs and green sea turtles provides a large-scale mechanism that enhances connectivity among seagrass meadows, and aids in resilience and recovery of these coastal habitats.

## Introduction

Dispersal is a critical stage in the life history of nearly all plant species, and limitations on this process may reduce connectivity between populations, lower resilience to natural and anthropogenic disturbances and inhibit recovery from large-scale declines due to propagule limitation^1, 2^. Although individual plant species may rely on one species-specific dispersal mechanisms (e.g. wind, rain)^3^, the majority of plant species use multiple dispersal mechanisms via both abiotic and biotic vectors^4, 5^.

Biotic dispersal of seeds by fauna occurs via bioturbation and the creation of drifting fragments that contain reproductive structures^6, 7^, the attachment of propagules to grazers (e.g. seeds stuck in waterfowl plumage) and subsequent deposition in a new location^8, 9^, or via direct consumption and excretion during grazing^10, 11^. Depending upon the size and mobility of the dispersal organism, seeds or plant propagules may be dispersed centimetres to kilometres^1–3^. For those seeds ingested during the dispersal process, the physical damage to seed coats during digestion and excretion may increase germination success through the process of scarification, or the splitting of the seed coat^4, 5^. This process alleviates physical dormancy imposed by the seed coat and initiates the germination process, serving as a primary germination cue for many terrestrial species^12^. Terrestrial plant-herbivore interactions are well studied, but little is known of these relationships in marine environments^10, 12, 13^.

Seagrasses are marine angiosperms which produce flowers, fruits and seeds almost entirely underwater^14^. Between 117,000 km^2^ and 500,000 km^2^ ^15, 16^ of seagrass meadows are found in shallow coastal waters around the world’s continents with the exception of Antarctica^15, 17, 18^. Within these habitats, seagrasses provide essential ecosystem functions including filtering nutrient run-off from terrestrial sources and stabilising marine sediment^19, 20^, providing nursery grounds for economically important fisheries species^21, 22^, and sequestering carbon^23, 24^. However, many seagrass meadows are under pressure from anthropogenic impacts (e.g. coastal development, fishing practices, agricultural and urban runoff)^16^ due to their proximity to areas of high human population^25, 26^. As a result of these stressors, approximately 7% of the known global area of seagrass is thought to be lost annually^16^. Many factors that influence seagrass meadow maintenance and recovery have been examined in both temperate and tropical habitats, but little is known concerning the modes of dispersal for these populations^1^.

The resilience of seagrass meadows is dependent on interactions between physical (e.g., location, climate, water currents and tidal flow) and biological factors (e.g. species and genetic diversity, life history strategy, population connectivity)^27–29^. Following large-scale losses of seagrass, seed germination from the sediment seed bank and subsequent seedling growth, is thought to be one of the main pathways for natural revegetation of disturbed habitats^10, 13^. Seed banks are replenished via seed production within the meadow and from the input of seeds, or propagules, from other more distant meadows. Deposition of seeds from outside sources results in more abundant seed banks and an increase in genetic diversity, culminating in an increased resilience to disturbance^28, 29^. However, most seagrass seeds are negatively buoyant. This inhibits dispersal by abiotic means unless seeds are attached to floating plant fragments^2, 14^. Significant knowledge gaps remain on mechanisms of seed dispersal in seagrass, levels of connectivity between meadows from seed dispersal^1, 2^, and particularly on the importance of biological dispersal vectors for maintaining the resilience and recovery capacity of seagrass meadows.

Research has primarily focused on abiotic dispersal of seagrass seeds and propagules^1, 29^. However, herbivores and omnivores in the marine environment, including crustaceans, echinoderms, fish, reptiles, birds, sea turtles and Sirenians (dugongs and manatees), consume seagrass either directly or indirectly while feeding^6, 10–12, 30^. As a result, fruiting bodies and seeds can be consumed, providing a potential for biotic dispersal^1^. Recent laboratory studies have shown that seeds of *Zostera marina* and *Halophila ovalis* can survive consumption by herbivorous fish and maintain or enhance their ability to germinate^31^. However, the ability of seeds to survive digestion by herbivores is species specific, as *Thalassia hemprichii* seeds did not survive consumption by waterfowl and fish under controlled laboratory conditions^9^. While these results indicate biotic dispersal of some seagrass by specific vectors is possible, there still remains a dearth of information on the role of animals, particularly mega-herbivores, in seagrass dispersal.

**Figure 1:**
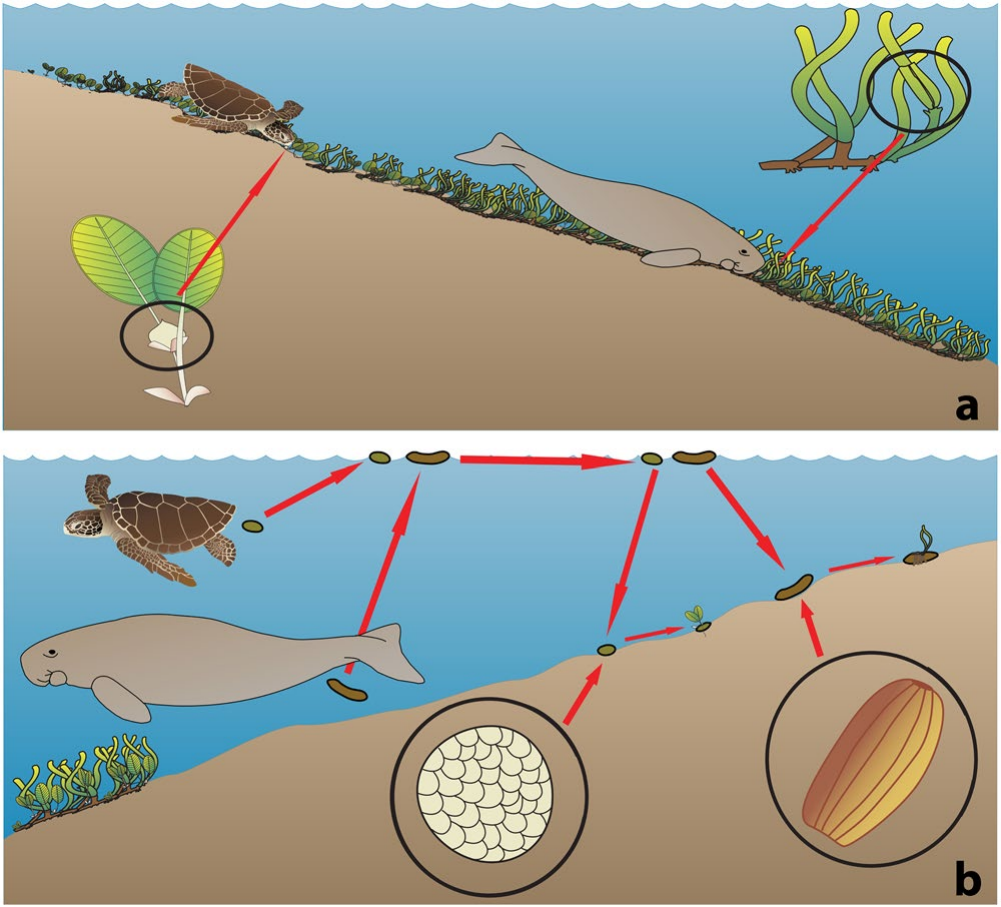
Illustration of biotic dispersal of seagrass seeds by marine mega- herbivores. (**a**) Ingestion of seagrass seeds or propagules by a dugong or green sea turtle allows long distance dispersal of the seed; (**b**) before settlement and growth into a seedling. Figure created by Samantha Tol, using images provided by Tracy Saxby, Catherine Collier, Diana Kleine, and the Integration and Application Network, University of Maryland Center for Environmental Science (ian.umces.edu/imagelibrary/).

In tropical habitats marine mega-herbivores, dugongs (*Dugong dugon*) and green sea turtles (*Chelonia mydas*), consume seagrasses in shallow coastal waters^32–34^. Dugongs conusme almost exclusively seagrass and include in their diet 24 out of the 26 seagrass species that co-occur in Australian waters^33^. Green sea turtles consume either a seagrass or algae dominated diet depending on their home range, however they will consume both food types when present^35^. Dugongs can consume up to 40 kg wet weight of seagrass daily^33, 36^ and green sea turtles consume up to 2.5% of their body weight in algae and/or seagrass daily^37^. Both marine mega-herbivores have digestion times ranging from several days to weeks; 6–8 days for dugongs and 7–14 days for green sea turtles (time variations are dependent on the digesta/food consumed, with a herbivorous diet taking longer to digest)^37–39^. Dugongs and green sea turtles actively move across their home ranges (green sea turtle median home range of 75.7 km^2^; dugong median home range of 453.2 km^2^)^40^ and can travel large distances when actively migrating (tagged dugongs have been observed to move at 25.9 ± 2.23 km per day when undertaking macro-scale movements, while green sea turtles can travel up to 40 km per day)^41–43^. The traits of high consumption rates, slow digestion and long distance movement combine to make dugongs and green sea turtles suitable vectors for long distance dispersal of seagrasses (Fig. 1).

For mega-herbivores in marine systems to disperse seagrasses, seeds must remain viable after passage through the gut; a factor not explored in previous studies. Our objectives in the present study were to (1) determine whether seagrass seeds pass through the digestive systems of marine mega-herbivores and remain viable and (2) determine the species, number, and potential dispersal distances for those seagrass species consumed by dugongs and green sea turtles in the Great Barrier Reef (GBR) Region. Our results quantify the potential for marine mega-herbivores to disperse seagrass seeds at different spatial scales and provide direct evidence of biotic dispersal by marine mega-herbivores.

## Results

### Faecal Samples

Marine mega-herbivore faecal samples collected floating on the water’s surface varied in size and mass; ranging from 0.002 g DW to 7.696 g DW, with an average of 1.37 ± 0.18 (SE) g DW (Supplementary Fig. S1).

### Seeds

**Figure 2:**
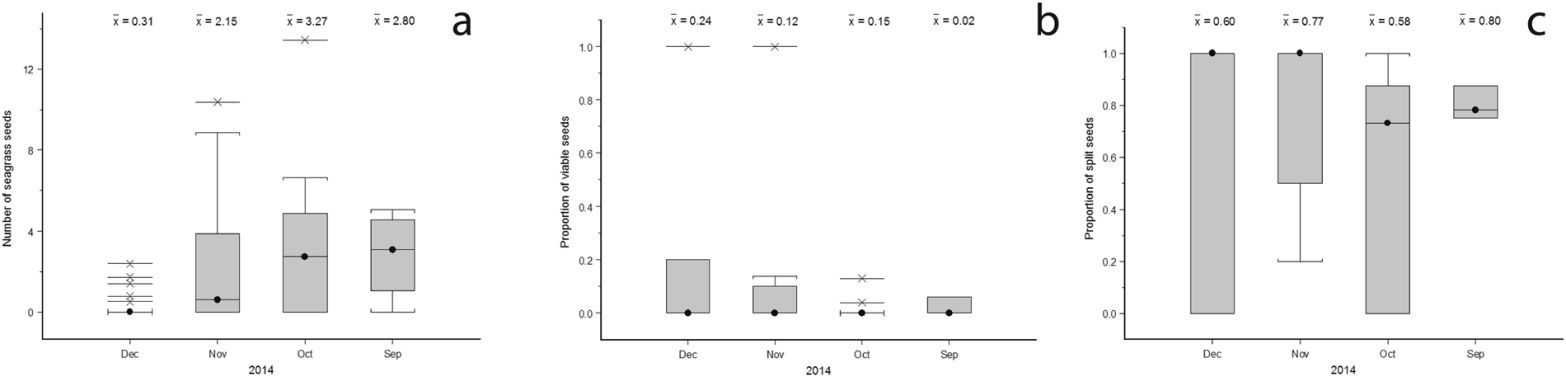
Mean monthly (**a**) number of seagrass seeds (*Zostera muelleri*, *Halodule uninervis*, *Halophila ovalis*, and *Halophila spp*.) per g DW mega-herbivore faecal matter; (**b**) proportion of seeds with a split seed coat per g DW faecal matter; (**c**) proportion of viable seagrass seeds found per g DW faecal matter collected across all sampling sites between September and December 2014. Crosses indicate data outliers for the sampled months and the box plots indicating the median, quartiles and 95 percentiles.

We found seagrass seeds in 56% of the marine mega-herbivore faecal samples. Seagrass seeds included *Zostera muelleri* (n = 219), *Halodule uninervis* (n = 3) *Halophila decipiens* (n = 7) and unidentified *Halophila spp*. (n = 4). The mean number of seagrass seeds was between 1–2 seeds per g DW faecal matter (Fig. 2a). The maximum density of seagrass seeds found within faecal samples were from Cleveland Bay (n = 153), followed by Pioneer Bay (n = 72), and Upstart Bay (n = 9). The month of December has significantly less seeds than the months of October (p = 0.034) and November (p = 0.019); seed abundance per sample (n = 8) is lowest in December, as is the percentage of faecal samples that contained seeds (September = 75.0%; October = 73.3%; November = 57.89%; December = 19.23%). Upstart Bay is significantly different to Pioneer Bay (p = 0.009) and Cleveland Bay (p = 0.055). Upstart Bay produced the least number of seeds (UB = 8; PB = 70; CB = 150) as well as the lowest percentage of faecal samples that contained seeds (UB = 30.0%; PB = 45.0%; CB = 60.0%).

**Figure 3:**
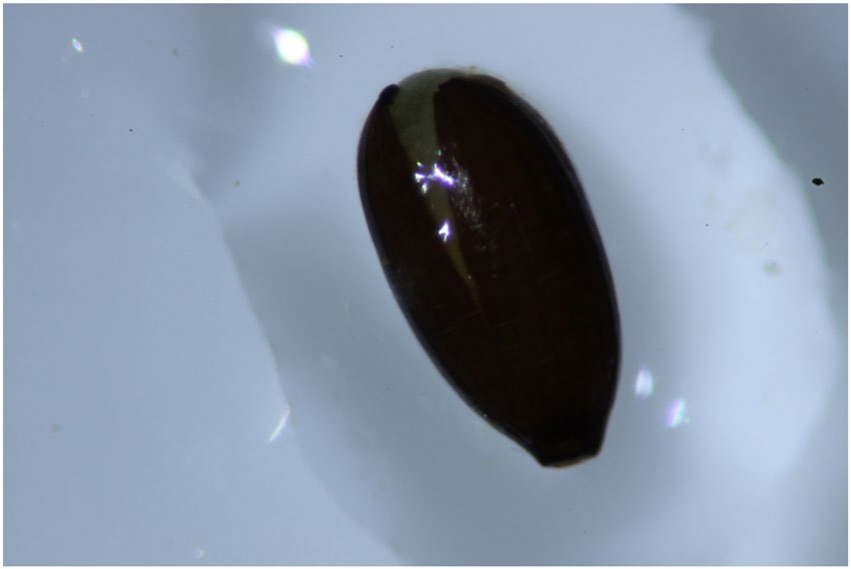
*Zostera muelleri* seed with a split coat; the seed was removed from a marine mega-herbivore faecal sample collected in Cleveland Bay, north Queensland Australia, in 2014.

Except for *H*. *decipiens*, all species of seagrass seeds found in faecal samples included at least one seed with a split coat (*Z*. *muelleri* split seeds = 39.73%; *Halodule uninervis* split seeds = 100%; *Halophila spp*. split seeds = 50%). Time and/or site of collection had no significant effect on the proportion of seeds which had their seed coat split (site p = 0.661; time p = 0.291) (Figs 2b and 3).

### Viability

Germination of seeds collected from mega-herbivore faeces occurred prior to viability testing for *Z*. *muelleri*, *Halophila decipiens* and *Halophila spp*., but not for *H*. *uninervis*. As germination is an indication of viability, these seeds were considered viable. Of the remaining non-germinated seeds, only *Z*. *muelleri* seeds tested as viable. The mean percentage of viable *Z*. *muelleri* seeds collected was 9.13% ± 4.61% (SE). Although the percentage of viable seeds was lower in samples collected in the earlier months (September and October) and peaked towards the end of the year (November and December), time of collection and/or site did not have a significant effect on the proportion of viable seeds (site p = 0.630; time p = 0.597) (Fig. 2c).

## Discussion

This is the first study to confirm the role of marine mega-herbivores, dugong﻿s (*Dugong dugon*) and green sea turtles *(Chelonia mydas)*, in the biotic dispersal of seagrass seeds. As the origin of the faecal samples (dugong or turtle) could not be accurately determined in the field, samples were combined into a single mega-herbivore category. We found 1–2 seeds per g DW in marine mega-herbivore faecal matter, with 9.13% ± 4.61% (SE) of these seeds maintaining viability after digestion. We found over half of the seeds excreted had a split seed coat, potentially increasing the chance of germination^44^. Time and site had only a minor effect on the number of seeds found per gram of marine mega-herbivore faeces. This is most likely due to different seagrass locations peaking in reproduction in the flowering season for these species.

For most seagrass species, long distance movement of reproductive structures (flowers, fruits and seeds) is possible only when these structures are attached to floating fragments^2, 29, 45, 46^. The potential movement of viable seeds by biotic dispersal, confirmed in our study, is an alternative and likely important pathway by which seagrass can colonise new or recovering locations. By identifying a potential long-distance dispersal vector for seagrasses, these results have direct implications for an increased understanding of the connectivity between seagrass meadows and existing natural resilience and recovery processes in seagrass meadows.

The dispersal capability of mega-herbivores depends on the availability of plant reproductive material, the number of mega-herbivores in the region, the amount of time the seagrass material stays within the digestive systems of herbivores and the potential distance travelled between consumption and excretion of the plant material^1, 2^. In the GBR the availability of viable seagrass reproductive material varies temporally, spatially and between species^47, 48^. In this study, maximum seed abundances in mega-herbivore faecal samples occurred in September and October while the greatest proportion of viable seeds were found in November and December. This reflects the dominance of *Z*. *muelleri* seeds in the samples and follows the observed periods of maximum flowering/reproductive density for *Z*. *muelleri* in this region^49^. In addition to its greater availably to mega-herbivores compared to other seagrass species across the sites sampled (Supplementary Table S2), the abundance of *Z*. *muelleri* seeds in the collected mega-herbivore faeces can be attributed to its flowering strategy. *Zostera muelleri* seeds are produced on branching flowering shoots that occur at the top of the meadow canopy^49^. The location and concentration of seeds above the sediment surface may make it easier for herbivores, green sea turtles in particular, to consume the seeds compared to species which flower at the base of the plant (e.g. *H*. *uninervis*)^50^. Therefore, the impacts of the timing and flowering shoot morphology on the biotic dispersal capability of seagrasses by mega-herbivores is likely to be species specific.

The GBR region supports a dugong population of at least 4,000–6,000 individuals^51, 52^ and an estimated population of 855,000 (95% CI: 55,000–1,200,000) green sea turtles (green sea turtle estimates based on best available data collected in the southern GBR)^53^. If these marine mega-herbivores conservatively pass one average size faeces per day, they have the potential to disperse as many as 500,000 viable seeds daily during the peak seagrass reproductive season (September to November). Based on current mega-herbivore population estimates, that would be an average of up to 2,500 viable seeds per day for dugongs and up to 500,000 viable seeds per day for green sea turtles. However, caution should be given when using these estimated seed dispersal numbers due to limitations in mega-herbivore abundance and migration data. Refined estimates of the number of individuals in the resident and migratory dugong and green sea turtle populations in the GBR, and the variability in the proportion of the turtle population that migrate into and out of the region, will be necessary to improve the accuracy of these dispersal estimates. However, the numbers presented here are likely to be conservative.

Due to the importance of connectivity in maintaining resilient seagrass meadows, it is important to quantify how many and how far seeds are dispersed^2, 54^. For biotic dispersal of seeds by mega-herbivores, dispersal distance is dependent on how far individual herbivores move while the seagrass material moves through their digestive system. Green sea turtles have a digesta retention time of 156–325 hours^38^, with an average travelling speed of 1.89 ± 0.12 km h^41, 42^, creating a potential dispersal distance of 277–652 km. However, most green sea turtles have a median home range of 75.7 km^2^ which may result in local rather than long-distance dispersal when turtles are not migrating^40^. Dugong gut passage times range from 146–166 hours^39^. They can travel on average at a speed of 1.3 ± 0.11 km h when swimming long distances^43^, potentially leading to a dispersal distance of 173–234 km; a distance less than the median home range of dugongs (453.2 km^2^)^40^. However, pedigree analysis in south-east Queensland found that only 1–3% of dugongs undertake largescale movements^55^, while recapture and telemetric data found long distance movement across foraging grounds is possible, with travel distances ranging up to 560 km^43, 55^. This suggests that dugongs have the potential to be long distance dispersers, however are more likely to disperse locally, similar to green sea turtles.

The actual distance travelled by seeds collected in this study cannot be measured. However, we found seeds in marine mega-herbivore faeces at one of our sites (Pioneer Bay) 100 s of kilometres from where seagrass flowering is common^48^, supporting the conclusion that these herbivores are an effective disperser of seagrass seeds. Distances shown in this study for seed dispersal, and previous studies on abiotic dispersal, indicate that there is a strong potential for the dispersal of seagrass among isolated meadows on reefs and islands and for the dispersal among countries by these mechanisms^29, 33, 56^.

Increases in the severity and occurrence of storms that have the ability to decimate seagrass meadows^20, 27, 57, 58^ have led to questions about the ability of seagrasses to re-establish or re-colonise after large losses^27, 29, 59^. Our present understanding of seagrass recovery mechanisms highlight the importance of the scale of the disturbance. Seagrass biomass loss on the scale of meters to tens of meters can recover primarily through rhizome extension^1, 2, 29, 45^. Seagrass expansion by clonal growth can occur quickly, allowing for rapid recovery from small scale impacts^2, 45^. However, clonal growth alone would be slow to re-colonise a large area if most seagrass biomass was lost^2^. To re-colonise a large area devoid of seagrass, input from either viable seagrass fragments or seeds would be required^2, 45^. Some coastal seagrass meadows have low connectivity to other meadows based on abiotic factors alone (such as wind and currents)^29^. Marine mega-herbivores may be vital for connectivity and gene flow among these meadows, as these animals are more likely to deposit seeds in habitats viable for seagrass growth compared to abiotic processes. The process of digestion aids in scarification, which through splitting the seed coat, removes physical dormancy and provides a cue for germination for some plant species^12^. Ultimately this may enhance germination rates and contribute to the recovery of impacted meadows. Deposition of genetically distinct seeds via mega-herbivore dispersal would increase the genetic diversity of meadows, thereby increasing their resiliency to disturbance events^60^.

Our study has confirmed biotic dispersal of viable seagrass seeds by marine mega-herbivores. As a result, tropical seagrass seeds have the potential to be dispersed far greater distances (in the hundreds of kilometres) than most previous reports suggest. The potential importance of mega herbivores in biotic dispersal is further enhanced by the large geographic distribution of these animals (green sea turtles from the tropical and sub-tropical ocean basins^56^ and dugongs from the east coast of Africa to the Indo-Pacific between latitudes 27 degrees north and south)^33^, which includes a large proportion of the world’s tropical and sub-tropical seagrass meadows. Biotic dispersal is also more likely to carry seeds to areas that are viable habitats for seagrass to grow, due to mega-herbivores actively searching for seagrass as a primary food source. This dispersal mode has the ability to contribute to the resilience of seagrass meadows and aid in recovery after loss. Our findings suggest that the conservation of green sea turtles and dugongs is likely to be far more important for maintaining the delicate balance between seagrass meadow recovery and loss than previously realised. A detailed understanding of the interconnection between marine mega-herbivores and their seagrass food is necessary to ensure a sustainable future for both these animals and their seagrass habitats.

## Methods

**Figure 4:**
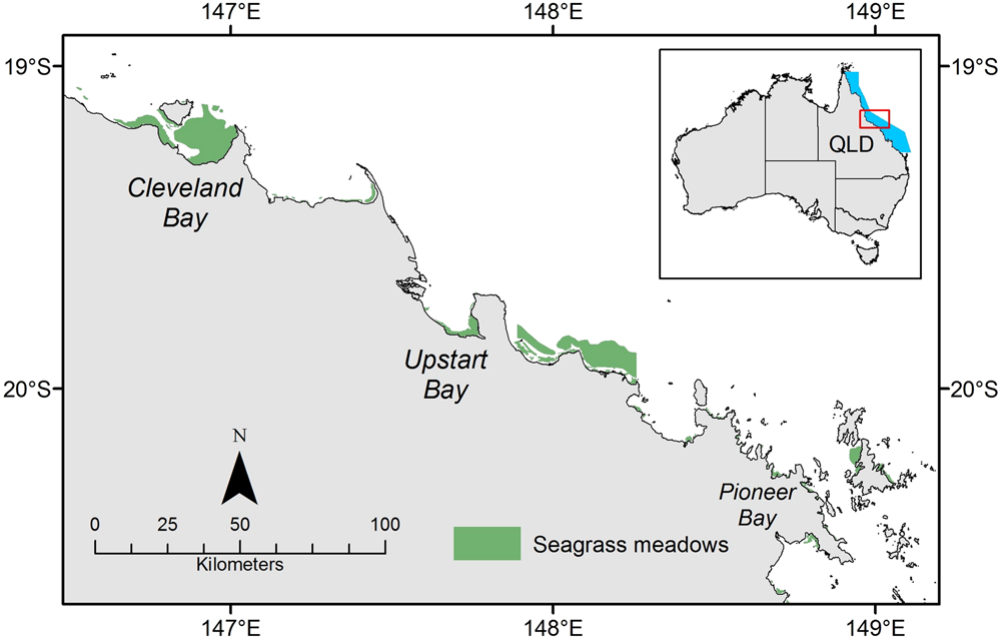
Map of intertidal seagrass meadows (green) in the central Great Barrier Reef, Queensland (QLD) Australia where mega-herbivore faecal matter samples were collected between September and December 2014. The inset map shows the location of the central region (red box) of the Great Barrier Reef World Heritage Area (blue). The map is overlayed with a subset of current known seagrass meadows (available from http://eatlas.org.au/data/uuid/77998615-bbab-4270-bcb1-96c46f56f85a). This map was created using ArcGIS v10.3 software by Esri software (http://www.esri.com/software/arcgis/new). ArcGIS and ArcMap are the intellectual property of Esri and are used herein under license.

Dugong and green sea turtle faecal samples were collected monthly between September and December 2014 from three coastal seagrass meadows in central GBR, Queensland; Pioneer Bay near the Whitsunday Island Group, Upstart Bay near the city of Bowen and Cleveland Bay near the city of Townsville (Fig. 4). Seagrass meadows at all sites consisted of mixed species assemblages and extended from the intertidal zone to the subtidal zone (Supplementary Table S2). Samples were collected during the period of seagrass maximum sexual reproduction, when 9 out of the 14 species present in the region flower^47^. During sample collections, green sea turtles were observed foraging at all sites, while dugongs were seen foraging at Pioneer Bay and Cleveland Bay. As it is not possible under field conditions to confidently determine from which of the two species the faeces had originated, and genetic differentiation was at the time outside the scope of this research, faecal samples from dugongs and green sea turtles were combined under a single ‘marine mega-herbivore’ category.

**Figure 5:**
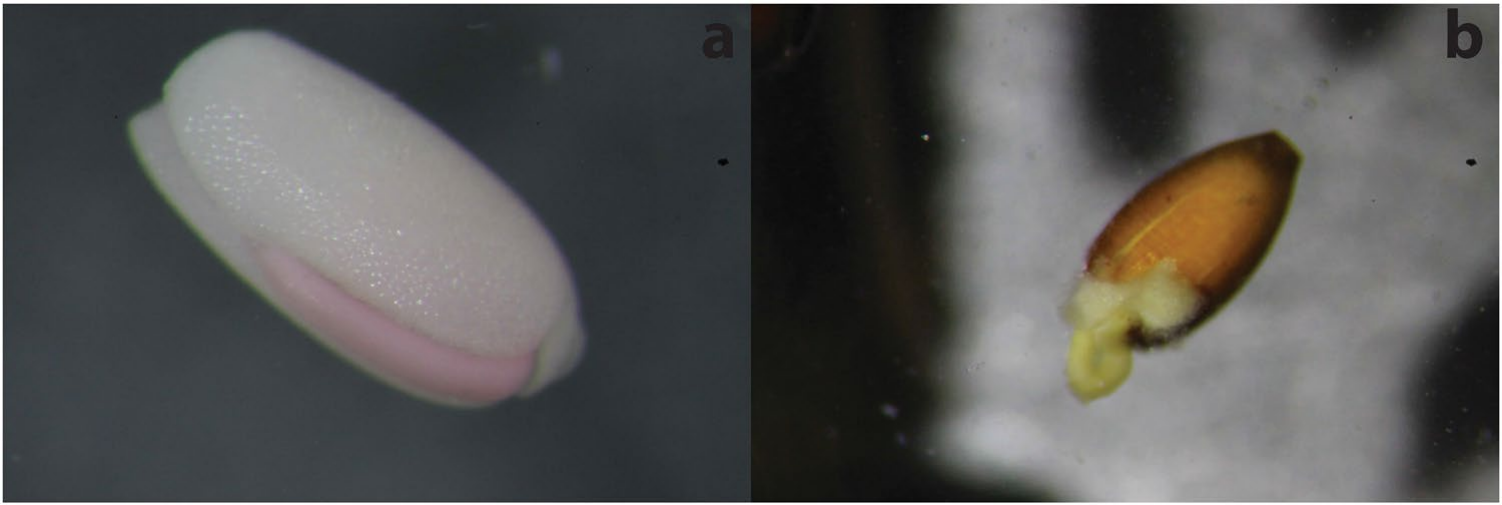
(**a**) A *Zostera muelleri* seed (with seed coat removed) taken from marine mega-herbivore faeces returned a positive stain, turning the cotyledon pink; (**b**) A germinated *Zostera muelleri* seed removed from marine mega-herbivore faeces.

A total of 60 faecal samples were collected over four months; 4 samples in September, 15 in October, 19 in November and 22 in December. Faecal samples were transported on ice to the laboratory where they were stored at 3–5 °C until processing. All samples were processed within 30 days of collection. Samples were sieved across three size fractions to aid in seed species identification (1.4mm, 750 µm and 250 µm) and then placed in 34 ± 1 PSU seawater to prevent osmotic stress^44^. Seagrass seeds found in faecal samples were identified to species and counted prior to the removal of the seed coat. Once the seed coat was removed, all non-germinated seeds were stained with a 0.5% Tetrazolium solution for a total of 48 hours to determine viability^44^. A positive stain (tissue turns brown to red in colour; Fig. 5a) indicated normal cellular metabolism within cells, signifying that the seed was still active and capable of germination^44, 61^. Any seeds removed from the faeces that had already germinated were counted as viable (Fig. 5b). Seeds with a split seed coat were also counted per sample. Faecal matter was dried at 220 °C in an oven until a consistent dry weight was reached. Samples were then weighed and reported as g DW.

Due to the low number of seeds collected per sample, seed data was combined across seagrass species and reported as the total number of seagrass seeds per g DW of faecal matter, the proportion of seeds with a split seed coat per g DW of faecal matter and the proportion of viable seagrass seeds per g DW of faecal matter (Fig. 2). Statistical analyses were conducted in the statistical software environment R (R version 3.2.5)^62^. Differences in the abundance of seagrass seeds per g DW of facecal matter over time (month) and site were analysed using generalized linear mixed effects models with a quasi-Poisson distribution (GLM)^62^; a Tukey post hoc test was applied. Quasi-Poisson regression is a generalized form of Poisson regression which corrects for overdispersion in count data^63^. Changes over time (month) and site in the proportion of seeds with a split seed coat and the proportions of seeds that stained as viable (positive stain + germinated seeds) per g DW of faecal matter were analyzed using logistic regression mixed effects models with a binomial distribution (GLM)^62^; a Tukey post hoc test was applied. Logistic regression was selected due to the binary response variable and the small number of seeds found per sample^64^.

### Data availability

The datasets generated during and/or analysed during the current study are available in the Tropical Data Hub repository; Citation: Tol, S. (2016). Biotic tropical seagrass seed dispersal by dugong and green sea turtles in the Great Barrier Reef. James Cook University. [Data Files] http://dx.doi.org/10.4225/28/57ABBA449E639, Digital Object Identifier (Doi):10.4225/28/57ABBA449E639.

## Electronic supplementary material

Supplementary material
