# Supplementary material for: Long distance biotic dispersal of tropical seagrass seeds by marine mega-herbivores
Source: Sci Rep. 2017 Jun 30;7:4458. doi: 10.1038/s41598-017-04421-1 (PMC5493642; doi:10.1038/s41598-017-04421-1)
Supplement: Supplementary file 1 — Supplementary material [file 41598_2017_4421_MOESM1_ESM.pdf]

Long distance biotic dispersal of tropical seagrass seeds by marine mega-herbivores

Tol. SJ[1, 2]*, Jarvis. JC[1, 3], York. PH[1], Grech. A[4], Congdon. BC[2] and Coles. RG[1]

1. Centre for Tropical Water and Aquatic Ecosystem Research (TropWATER), James Cook University, Cairns Australia;
2. College of Science and Engineering, James Cook University, Cairns Australia;
3. University of North Carolina Wilmington, United States of America
4. ARC Centre of Excellence for Coral Reef Studies, James Cook University, Townsville, Australia.

Supplementary Figure S1: a) Marine mega-herbivore faeces collected floating on the water's surface in Pioneer Bay at Airlie Beach in the Whitsundays, northern Queensland Australia; **b)** Floating marine mega-herbivore faeces floating on the water's surface. Picture taken at Pioneer Bay in the Whitsunday Island Group, northern Queensland Australia.

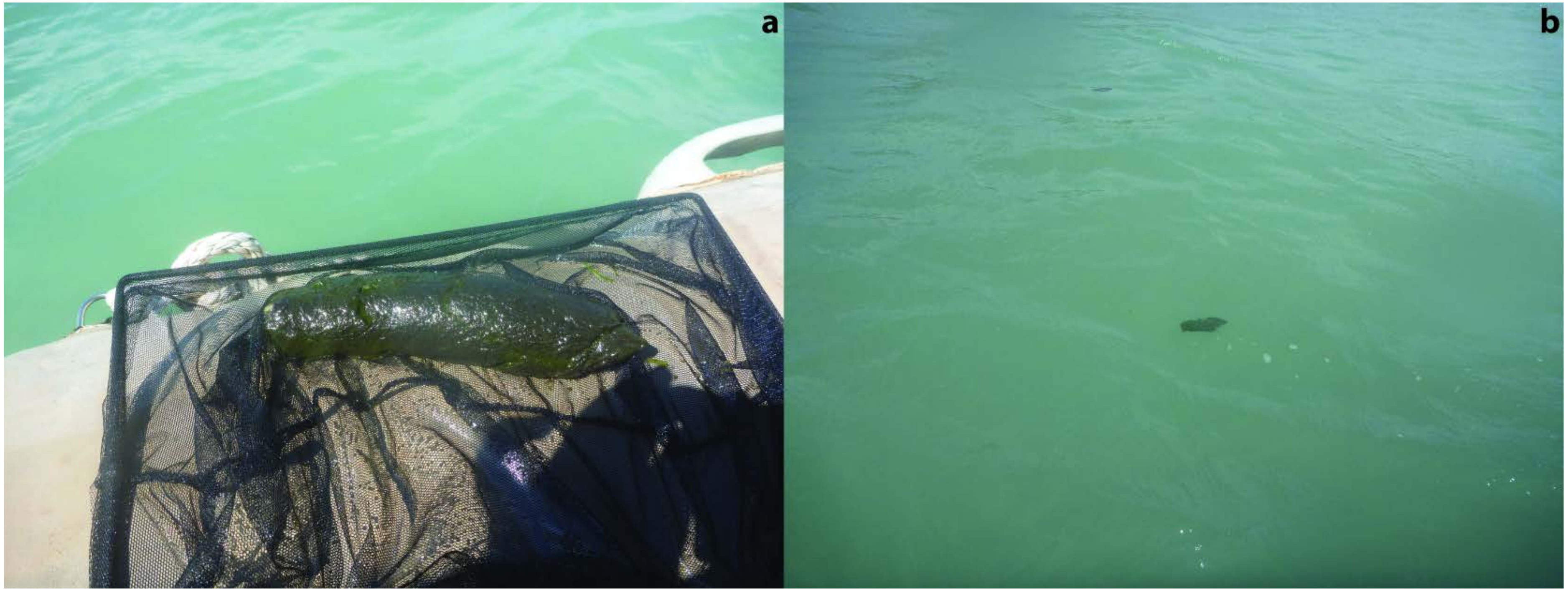

Supplementary Table S2: Description of intertidal seagrass meadows in northern Queensland, Australia; Pioneer Bay in the Whitsunday Island Group, Upstart Bay near the city of Bowen and Cleveland Bay near the city of Townsville.

Location	Site Description	Species Present	Dominant Species	Total Mean Above Ground Biomass (gdw m^2)	Total area (ha)
Pioneer Bay [1,2]	Sand/mud sediment	*Halophila spp.*<br>*Halophila ovalis*<br>*Halodule uninervis*<br>*Zostera muelleri*	*Zostera muelleri* / *Halodule uninervis*	0.59 ±0.1	141.1 ±40
Upstart Bay [3]	Mud/sand sediment	*Cymodocea serrulata*<br>*Halophila decipiens*<br>*Halophila ovalis*<br>*Halophila spinulosa*<br>*Halophila tricostata*<br>*Halodule uninervis*	*Zostera muelleri*	21.0 ±5.3	2987 ±532

		Zostera muelleri			
Cleveland Bay [4]	Sand/mud sediment	*Cymodocea serrulata*<br>*Halophila decipiens*<br>*Halophila ovalis*<br>*Halophila spinulosa*<br>*Halodule uninervis*<br>*Zostera muelleri*	*Zostera muelleri* (Intertidal)<br>*Halodule uninervis* (Subtidal)	10.98 ±1.06 (Intertidal)<br>6.34 ±0.46 (Subtidal)	1479.81 ±101.3 (Intertidal)<br>4016.85 ±438.9 (Subtidal)

1 Campbell, S., Roder, C., McKenzie, L. & Lee Long, W. Seagrass Resources in the Whitsunday Region 1999 and 2000. 50 (Department of Primary Industries, Cairns, 2002).

2 McKenzie, L. Seagrass-Watch Whitsundays (Seagrass-Watch, 2015). At <http://www.seagrasswatch.org/whitsundays.html>

3 Coles, R., Lee Long, W., McKenzie, L. & Roder, C. Seagrass and Marine Resources in the Dugong Protection Areas of Upstart Bay, Newry Region, Sand Bay, Llewellyn Bay, Ince Bay and the Clairview Region April/May 1999 and October 1999. 141 (Marine Plant Ecology Group, Northern Fisheries Centre, Department of Primary Industries, Cairns, Queensland, 2002).

4 Davies, J., McKenna, S. & Rasheed, M. Port of Townsville long term seagrass monitoring: September 2012. 32 (Centre for Tropical Water & Aquatic Ecosytem Research (TropWATER) Publication, James Cook University, Cairns, Australia, 2013).
